# Inhibition of XPO1 impairs cholangiocarcinoma cell proliferation by triggering p53 intranuclear accumulation

**DOI:** 10.1002/cam4.5322

**Published:** 2022-10-05

**Authors:** Cheng Zhao, Ben Ma, Zi‐yi Yang, Ou Li, Shi‐lei Liu, Li‐jia Pan, Wei Gong, Ping Dong, Yi‐jun Shu

**Affiliations:** ^1^ Laboratory of General Surgery and Department of General Surgery Xinhua Hospital affiliated with Shanghai Jiao Tong University School of Medicine Shanghai China; ^2^ Shanghai Key Laboratory of Biliary Tract Disease Research Shanghai China

## Abstract

**Background:**

XPO1 mediates the nuclear export of several proteins, mainly tumor suppressors. KPT‐330 (Selinexor) is a selective inhibitor of XPO1 that has demonstrated good therapeutic effects in hematologic cancers.

**Methods:**

We used TCGA and GTEx pan‐cancer database to evaluate XPO1 mRNA expression in various tumors. Cell proliferation assay and colony formation assay were used to analyze the in vitro antitumor effects of XPO1 inhibitor KPT‐330. Western blot was performed to explore the specific mechanisms.

**Results:**

We found that XPO1 was highly expressed across a range of cancers and associated with poor prognosis in hepatobiliary and pancreatic tumors. We revealed that the XPO1 inhibitor KPT‐330 triggered the nuclear accumulation of the p53 protein and significantly disrupted the proliferation of cholangiocarcinoma cells. Mechanistically, the XPO1 inhibitor, KPT‐330, reduced BIRC6 expression by inhibiting the PI3K/AKT pathway to decrease p53 degradation and improve its stability.

**Conclusion:**

Therefore, XPO1 may be a potential therapeutic target in cholangiocarcinoma, mediated by its effects on KPT‐330.

## Graphical abstract

We found that XPO1 showed high expression in pan‐cancer and was associated with poor prognosis in hepatobiliary and pancreatic tumors. In CHOL, we revealed that XPO1 inhibitor KPT‐330 could trigger the nuclear accumulation of the p53 protein and in this way significantly disrupted the proliferation of cholangiocarcinoma cells. Mechanistically, XPO1 inhibitor KPT‐330 reduced BIRC6 expression by inhibiting PI3K/AKT pathway to decrease p53 degradation and improve its stability

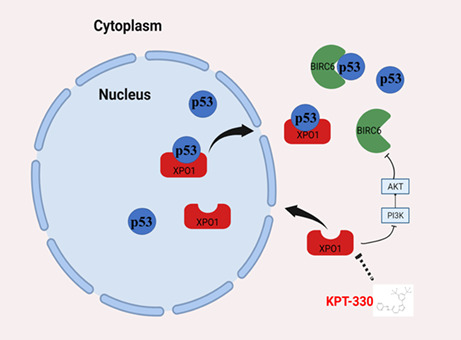

**Abbreviations**
XPO1: Exportin‐1
FDA: United States Food and Drug Administration
BLCA: Bladder Urothelial Carcinoma
BRCA: Breast invasive carcinoma
CESC: Cervical squamous cell carcinoma and endocervical adenocarcinoma
CHOL: Cholangiocarcinoma
COAD: Colon adenocarcinoma
DLBC: Lymphoid Neoplasm Diffuse Large B‐cell Lymphoma
ESCA: Esophageal carcinoma
GBM: Glioblastoma multiforme
HNSC: Head and Neck squamous cell carcinoma
KICH: Kidney Chromophobe
KIRP: Kidney renal papillary cell carcinoma
LAML: Acute Myeloid Leukemia
LGG: Brain Lower Grade Glioma
LIHC: Liver hepatocellular carcinoma
LUAD: Lung adenocarcinoma
LUSC: Lung squamous cell carcinoma
OV: Ovarian serous cystadenocarcinoma
PAAD: Pancreatic adenocarcinoma
READ: Rectum adenocarcinoma
STAD: Stomach adenocarcinoma
TGCT: Testicular Germ Cell Tumors
THCA: Thyroid carcinoma
THYM: Thymoma
TCGA: The Cancer Genome Atlas
KEGG: Kyoto Encyclopedia of Genes and Genomes
CHX: cycloheximide

## 1 BACKGROUND

Cholangiocarcinoma is a highly aggressive malignancy, characterized by cholangiocyte differentiation.^1^ The 5‐year survival rate for cholangiocarcinoma is 20%–40%, however, the only effective treatment is complete surgical resection.^2^ Unfortunately, owing to the lack of typical clinical manifestations, many patients are diagnosed at an advanced stage and are not eligible for surgery.^3^ Therefore, there is an urgent need to identify new diagnostic and therapeutic targets for the treatment of cholangiocarcinoma.

Exportin‐1 (XPO1) is a major nuclear export receptor protein that transports cargo proteins via a leucine‐rich nuclear export signal (NES) from the nucleus to the cytoplasm.^4^ Cargo proteins of XPO1 are mainly tumor suppressors, including p21 and p53.^5^ High expression of XPO1 has been reported in various tumors, including pancreatic adenocarcinoma, gastric cancer, neuroblastoma, and hematologic tumors, and is strongly associated with poor prognosis.^6^, ^7^, ^8^, ^9^ Therefore, the inhibition of XPO1 may be a therapeutic tool for tumors. Scientists have developed selective inhibitors for XPO1 over the years, and Selinexor (KPT‐330) has been clinically validated and approved by the United States Food and Drug Administration.^10^ However, the role of XPO1 and its therapeutic effect on KPT‐330 on cholangiocarcinoma has yet to be elucidated.

In this study, we analyzed the information in the Cancer Genome Atlas (TCGA) database and found that XPO1 was highly expressed in 21 types of tumors and was clearly associated with poor patient prognosis. We used bioinformatics analysis to clarify that the genes positively associated with high expression of XPO1 were mainly associated with ubiquitin‐mediated proteolysis, RNA transport, and the spliceosome. We found that XPO1 inhibition by KPT‐330 significantly disrupted cholangiocarcinoma cell proliferations. Further experiments revealed that the XPO1 inhibitor KPT‐330 increased intranuclear accumulation of p53 and increased its protein stability. We discovered that BIRC6 could cause p53 degradation; however, KPT‐330 reduced BIRC6 expression by inhibiting PI3K/AKT pathway to alleviate p53 degradation. Therefore, XPO1 may be a potential therapeutic target and KPT‐330 may play a therapeutic role in cholangiocarcinoma.

## 2 MATERIALS AND METHODS

### 2.1 Cell culture and treatment

Cholangiocarcinoma cells (RBE and 9810) were purchased from the Cell Bank of the Shanghai Institute for Biological Sciences, Chinese Academy of Sciences. Cells were cultured in DMEM medium (Gibco) supplemented with 10% fetal bovine serum (Gibco) in a humid chamber at 37°C with 5% CO2. KPT‐330, cycloheximide (CHX), and 740 Y‐P were purchased from Selleck Chemicals.

### 2.2 RNA extraction and qRT‐PCR

Total RNAs were extracted from RBE and 9810 cells by using Trizol reagent (Invitrogen). cDNA was generated using the PrimeScript RT reagent kit with gDNA Eraser (TaKaRa) according to the manufacturer's instructions. The primers used for amplification were followed: GAPDH forward primer (5‐CAACAGCCTCAAGATCATCAGC‐3), GAPDH reverse primer (5‐TTCTAGACGGCAGGTCAGGTC‐3), p53 forward primer (5‐ CAGCACATGACGGAG GTTGT‐3), and p53 reverse primer (5‐TCATCCAAA TACTCCACACGC‐3).

### 2.3 Western blot

Total protein extraction and western blotting were conducted as previously described.^11^ In brief, first, proteins were isolated with RIPA Lysis buffer (Beyotime). Next, proteins were separated by SDS‐PAGE and transferred onto PVDF membranes (Millipore). Then, 5% skim milk was used to block the blots for 1 h at room temperature. A series of primary antibodies (Abcam) was added to the appropriate position of the PVDF membranes and incubated overnight at 4°C. Finally, all blots reacted with the suitable Horseradish Peroxidase‐conjugated secondary antibody (Beyotime), and the immunoreactive bands were detected by chemiluminescence and visualized using a Gel Doc 2000 (Bio‐Rad). Antibodies against XPO1, p53, H3, BIRC6, PI3K, p‐PI3K(Tyr458), AKT, p‐AKT(Ser473), and GAPDH were purchased from Abcam.

### 2.4 Cell proliferation assays

Approximately 1500 cells of RBE and 9810 cells were seeded in 96‐well plates. Cell proliferation was assessed using a Cell Counting Kit‐8 assay (Yeasen). The cell proliferation curves were plotted using absorbance at 450 nm.

### 2.5 Colony formation assay

RBE and 9810 cells were seeded in 6‐well plates at a density of 1000 cells per well for 10 days. The cells were fixed with 4% paraformaldehyde and stained with 0.1% crystal violet.

### 2.6 siRNA, plasmid construction, and transfection

The siRNA‐transfected cholangiocarcinoma cells used were as follows: p53‐siRNA: 5‐CGGCGCACAGAGGAAGAGAAUTT‐3. R‐fect (Baidai) was used to transfect siRNA. The plasmid pBIRC6, which encodes the full‐length cDNA of the BIRC6 (NM_001378125.1) and the XPO1 (NM_001410799.1) was constructed by Genomeditech. The empty vector (pCMV6) was used as a negative control. Viafect transfection reagent was used for plasmid transfection according to the protocol (Promega).

### 2.7 Immunofluorescence assay

RBE and 9810 cells were seeded on coverslips 1 day before and fixed using 4% paraformaldehyde for 15 min at room temperature. We implied Immunofluorescence Staining Kit (Beyotime) according to the protocol. A fluorescence microscope (Leica DM4B) was used to capture images.

### 2.8 Data collection and analysis

XPO1 expression profiles and TCGA and Genotype‐Tissue Expression (GTEx) clinical pan‐cancer data were downloaded from the University of California, Santa Cruz (UCSC) Xena database (https://xenabrowser.net/datapages/). For pan‐cancer data of TCGA and GTEx databases, differential XPO1 mRNA expression between various tumors and normal tissues was analyzed using *t* test, and their visualization was carried out by R software package “ggplot2.” For data transformation, RNAseq data in Fregments Per Kilobase per Million (FPKM) format were converted to transcripts per million reads (TPM) format and log2 transformed. In order to assess the expression of XPO1, tumor tissues were obtained from TCGA, and normal tissues were obtained from TCGA and the GTEx databases.

### 2.9 Correlation and enrichment analysis

Pearson correlation analysis of XPO1 mRNA and other mRNAs was conducted in cholangiocarcinoma using TCGA CHOL data. The 500 genes most positively associated with XPO1 were selected for enrichment analysis to determine the function of XPO1. Gene ontology (GO) analysis was performed using the EnrichGO function in the *clusterProfiler* R software package R with the following parameters: *p* value.adj <0.1 and *Q* value <0.2.

### 2.10 Statistical analysis

Statistical analyses were performed with Prism 8 (GraphPad Software) and SPSS 24 (SPSS Inc.). Student's *t* test was performed between two groups, and analysis between multiple groups was conducted by one‐way analysis of variance, data are recorded in the form of mean ± SD. *p* Values of <0.05 were considered statistically significant (*p < 0.05, **p < 0.01, ***p < 0.001).

## 3 RESULTS

### 3.1 Pan‐cancer expression of XPO1

**FIGURE 1:**
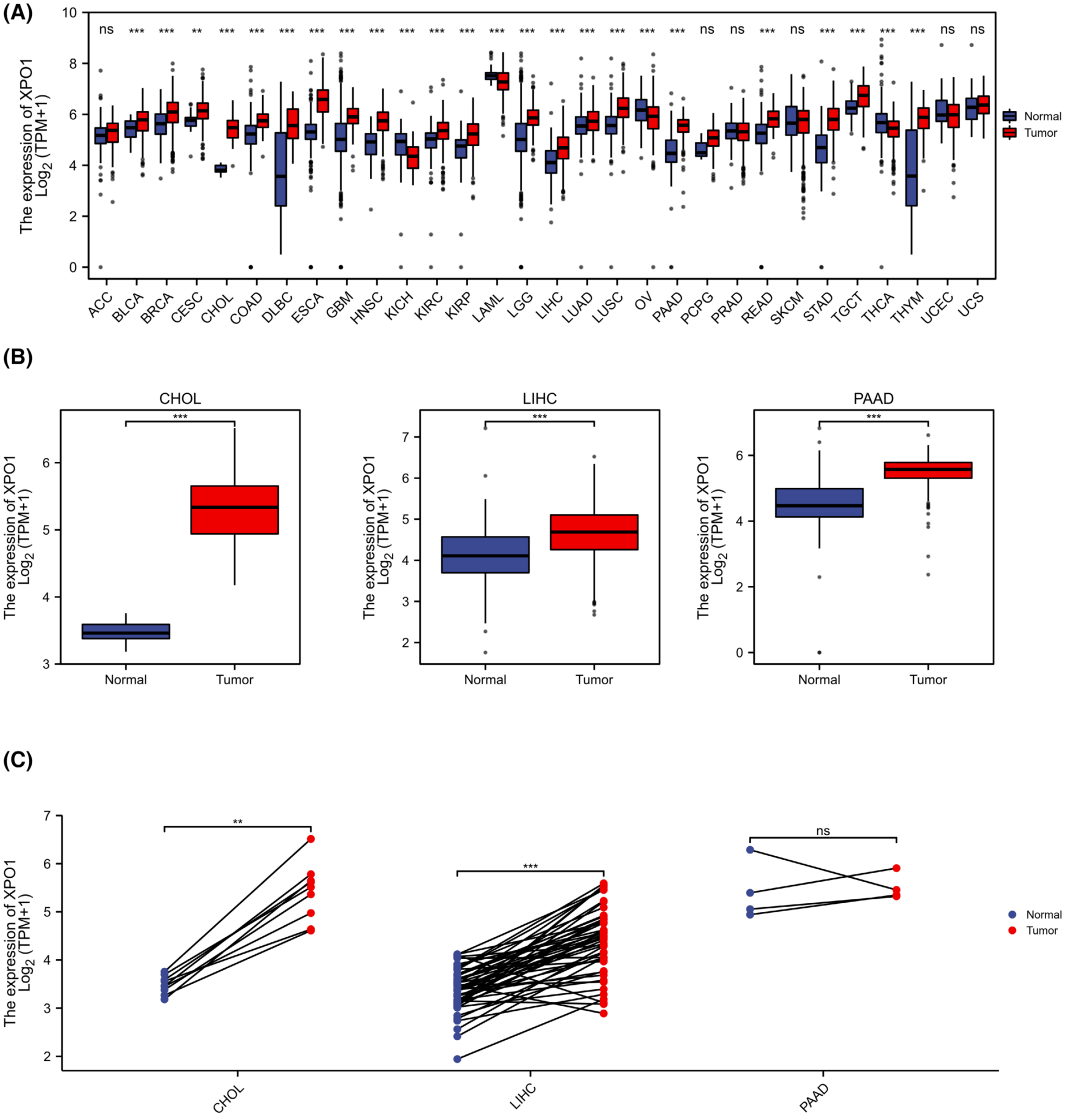
Pan‐cancer expression of XPO1. (A) XPO1 expression in tumor and normal tissues in pan‐cancer data of the Cancer Genome Atlas (TCGA) and GTEx. (B) XPO1 expression in tumor and normal tissues in CHOL, LIHC, and PAAD from TCGA. (C) XPO1 expression in paired tumor and normal tissues in CHOL, LIHC, and PAAD from TCGA. Data were shown as mean ± SD. *p < 0.05, **p < 0.01, ***p < 0.001, and ****p < 0.0001. CHOL, cholangiocarcinoma; GTEx, genotype‐tissue expression; LIHC, liver hepatocellular carcinoma; PAAD, pancreatic adenocarcinoma; TCGA, the cancer genome atlas; XPO1, exportin‐1.

Using TCGA and GTEx pan‐cancer database, we evaluated XPO1 mRNA expression in various tumors. The results of the analysis suggested that XPO1 showed high expression in 21 types of tumors, including bladder cancer (BLCA), breast cancer (BRCA), cervical cancer (CESC), cholangiocarcinoma (CHOL), colon adenocarcinoma (COAD), diffuse large B‐cell lymphoma (DLBC), esophageal carcinoma (ESCA), glioblastoma (GBM), head and neck squamous cell carcinoma (HNSC), kidney chromophobe (KICH), kidney renal papillary cell carcinoma (KIRP), acute myeloid leukemia (LAML), brain lower grade glioma (LGG), liver hepatocellular carcinoma (LIHC), lung adenocarcinoma (LUAD), lung squamous cell carcinoma (LUSC), pancreatic adenocarcinoma (PAAD), rectum adenocarcinoma (READ), stomach adenocarcinoma (STAD), testicular germ cell tumors (TGCT), and thymoma (THYM) (Figure 1A). Notably, in TCGA unpaired samples, XPO1 was highly expressed in hepatobiliary and pancreatic tumors (CHOL, LIHC, and PAAD) (Figure 1B). In the paired samples, we found that XPO1 was highly expressed in CHOL and LIHC compared with the corresponding normal tissues (pancreatic cancer pairing data lacked significance with only four pairs) (Figure 1C). These results suggest that XPO1 may play an important role in tumor development and warrant further investigation.

### 3.2 Association between high XPO1 expression and tumor prognosis in patients

**FIGURE 2:**
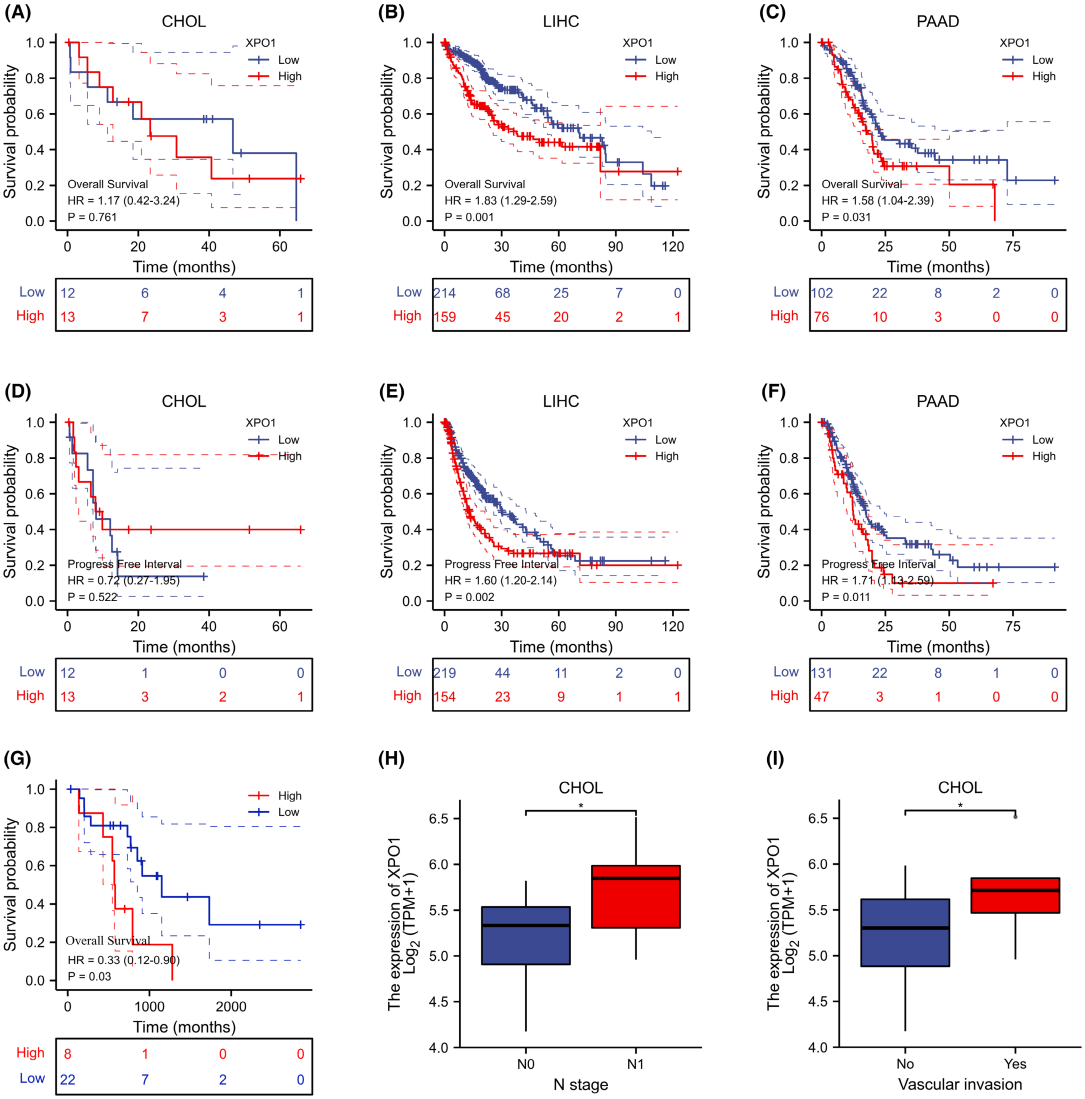
Association between high XPO1 expression and tumor prognosis in patients. (A–F) The correlation between XPO1 expression and the prognosis of CHOL, LIHC, and PAAD was analyzed using TCGA. (G) The correlation between XPO1 expression and the prognosis of CHOL was analyzed using the GEO database. (H) The correlation between XPO1 expression and “N stage” in CHOL. (I) The correlation between XPO1 expression and “Vascular invasion” in CHOL. Data were shown as mean ± SD. *p < 0.05, **p < 0.01, ***p < 0.001, ****p < 0.0001. CHOL, cholangiocarcinoma; LIHC, liver hepatocellular carcinoma; PAAD, pancreatic adenocarcinoma; TCGA, the cancer genome atlas; XPO1, exportin‐1.

Next, we focused on hepatobiliary and pancreatic tumors: CHOL, LIHC, and PAAD. To further investigate the relationship between high XPO1 expression and patient prognosis, we analyzed its association with “overall survival” and “progress‐free interval.” As shown in Figure 2A–F, higher XPO1 expression was significantly related to poor patient prognosis in LIHC and PAAD; however, we did not observe a significant difference in CHOL. We analyzed the prognosis of 30 patients with cholangiocarcinoma using GSE107943 from the GEO database and the results showed that higher expression of XPO1 was significantly associated with poor patient prognosis in CHOL (Figure 2G). In the data on “N stage” and “vascular invasion,” we found significant differences in CHOL (Figure 2H,I). These results suggest that high XPO1 expression in hepatobiliary and pancreatic tumors is strongly associated with poor prognosis.

### 3.3 Correlation and enrichment analyses in CHOL

**FIGURE 3:**
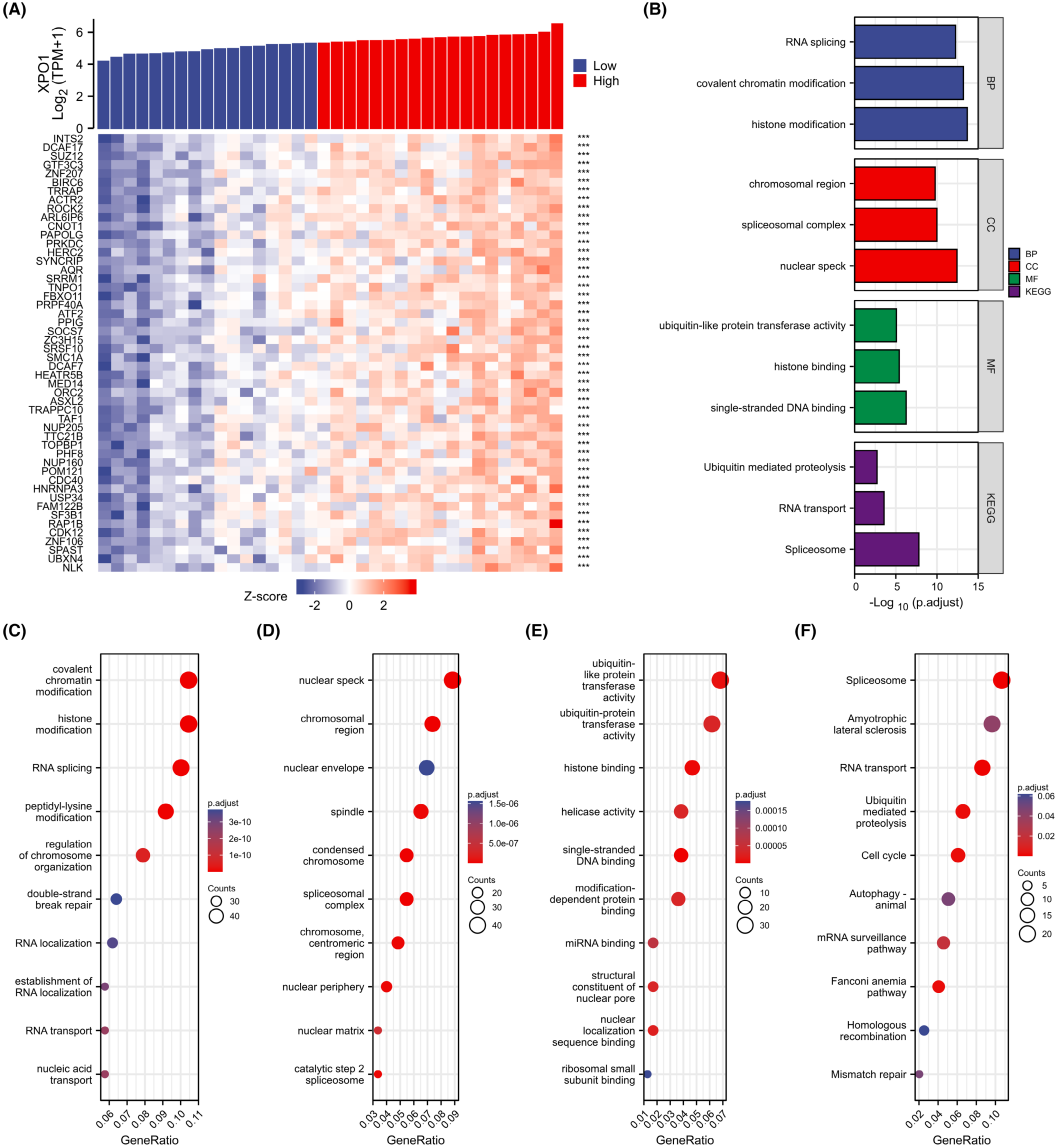
Correlation and enrichment analyses in CHOL. (A) The top 50 genes positively correlated with XPO1in a heat map (ranked according to correlation) from TCGA. (B–F) Significant Gene Ontology terms of the top 500 genes most positively associated with XPO1, including biological processes (C), cell component (D), molecular function (E), and KEGG (F). CHOL, cholangiocarcinoma; KEGG, Kyoto encyclopedia of genes and genomes; TCGA, the cancer genome atlas; XPO1, exportin‐1.

To further elucidate the function of XPO1 in CHOL, we analyzed the genes that were positively correlated with XPO1 expression in the TCGA database. We selected the 500 genes that were most positively correlated with XPO1 for enrichment analysis and displayed the top 50 genes in a heat map (ranked according to correlation) (Figure 3A). Furthermore, we used the R clusterProfiler package to analyze the possible enrichment pathways and correlation of these 500 genes. GO functional enrichment analysis indicated that XPO1 was related to modified and binding functions, which corresponded to the transport function of XPO1 (Figure 3B–E). The Kyoto Encyclopedia of Genes and Genomes (KEGG) analysis revealed that spliceosome, RNA transport, ubiquitin‐mediated proteolysis, and cell cycle terms were significantly enriched (Figure 3A,F). These results indicate that high XPO1 expression is associated with multiple pathways related to cholangiocarcinoma progression which is closely linked to XPO1 transport function.

### 3.4 Effect of the XPO1 inhibitor KPT‐330 on cholangiocarcinoma cell proliferation

**FIGURE 4:**
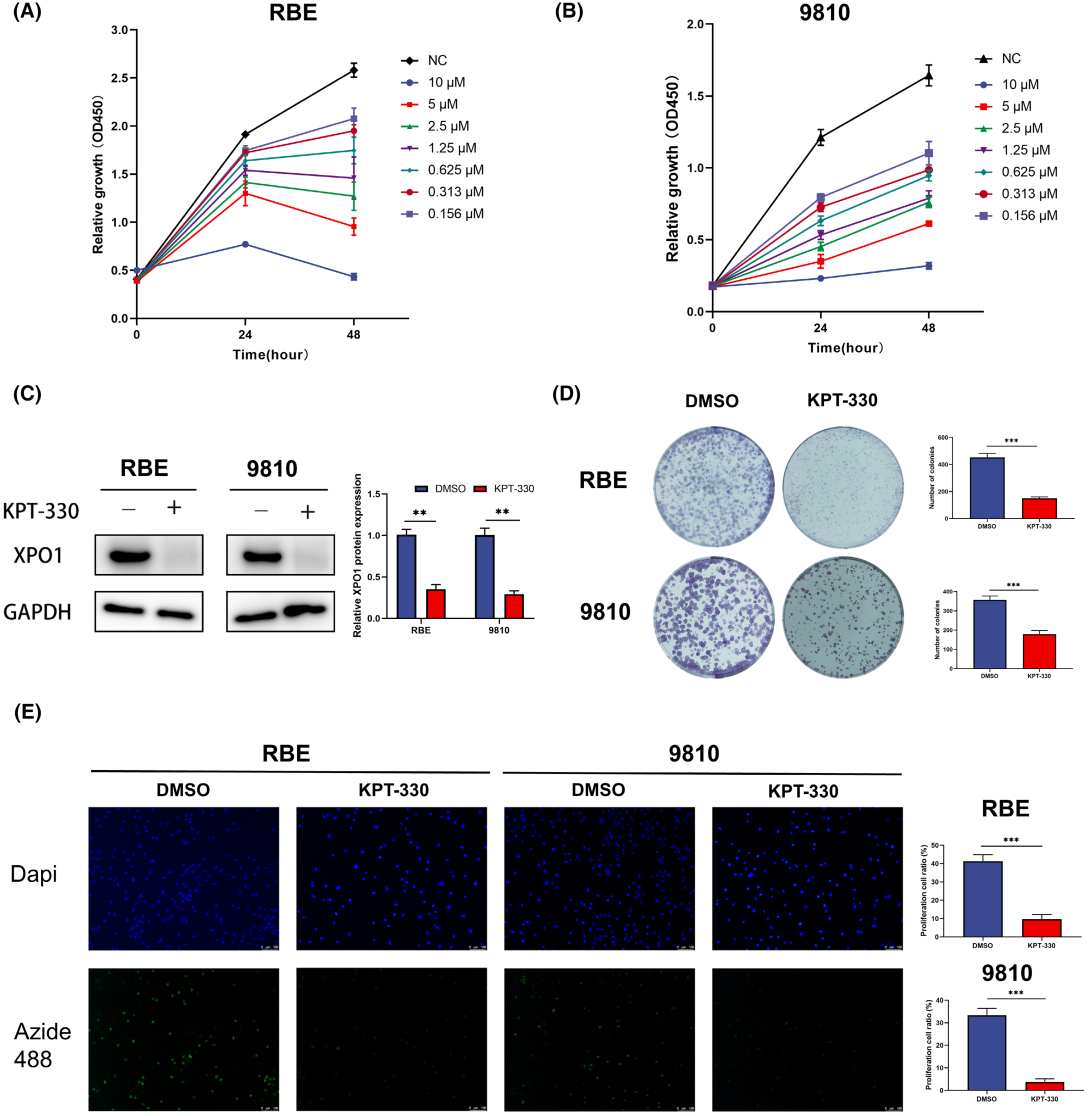
Effect of exportin‐1 (XPO1) inhibitor KPT‐330 on cholangiocarcinoma cell proliferation. (A, B) Cell proliferation of RBE and 9810 under KPT‐330 treatment. (C) Western blot of XPO1 under KPT‐330 treatment. (D) The colony formation of RBE and 9810 under KPT‐330 treatment. (E) EdU‐488 DNA synthesis assay of RBE and 9810 under KPT‐330 treatment. Data were shown as mean ± SD. *p < 0.05, **p < 0.01, ***p < 0.001, ****p < 0.0001.

Based on bioinformatics analysis, we performed basic experiments to verify the effect of XPO1 on cholangiocarcinoma tumor cells. We found that the proliferation of RBE and 9810 cells was inhibited by the XPO1 inhibitor KPT‐330 in time‐ and concentration‐dependent manners (Figure 4A,B). We chose a KPT‐330 concentration of 2.5 μM and a treatment time of 48 hours for subsequent experiments. We conducted a western blot and found that XPO1 was effectively inhibited by KPT‐330 in RBE and 9810 cells (Figure 4C). The colony formation assay indicated that KPT‐330 significantly reduced the number and size of colonies formed by cholangiocarcinoma cells (Figure 4D), further confirming its antiproliferative effect. Further, EdU‐488 DNA synthesis assay experiments showed the inhibitory effect of KPT‐330 on the proliferation of RBE and 9810 (Figure 4E). These results indicate that XPO1 promotes cholangiocarcinoma cell proliferation.

### 3.5 Overexpression of XPO1 promoted cholangiocarcinoma cell proliferation

**FIGURE 5:**
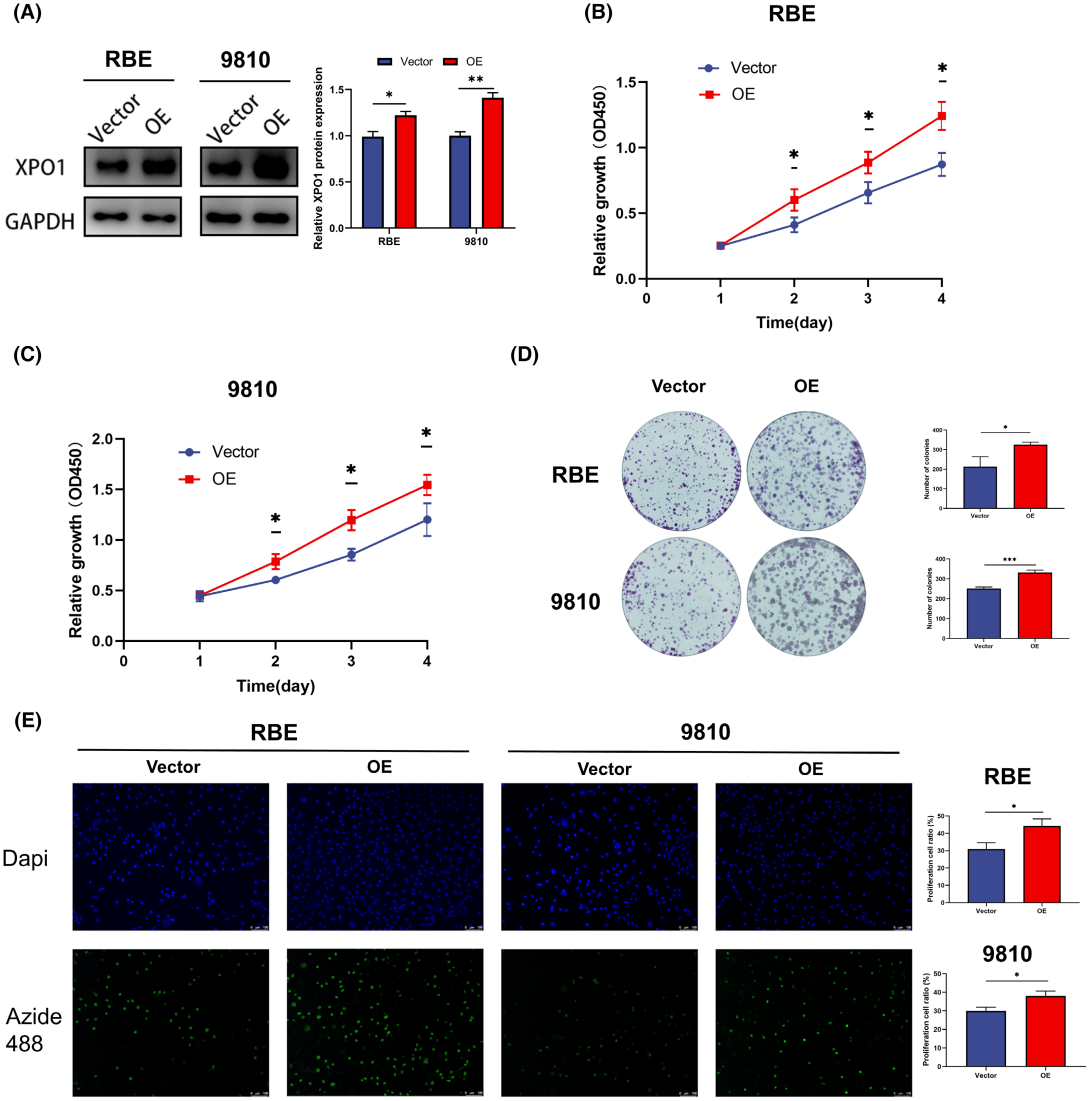
Overexpression of exportin‐1 (XPO1) promoted cholangiocarcinoma cell proliferation. (A) Western blot of XPO1 after XPO1 plasmid transfection in RBE and 9810 cells. (B) Cell proliferation of RBE after XPO1 plasmid transfection. (C) Cell proliferation of 9810 after XPO1 plasmid transfection. (D) The colony formation of RBE and 9810 after XPO1 plasmid transfection. (E) EdU‐488 DNA synthesis assay of RBE and 9810 after XPO1 plasmid transfection. Data were shown as mean ± SD. *p < 0.05, **p < 0.01, ***p < 0.001, ****p < 0.0001.

We used XPO1 overexpression plasmid in RBE and 9810 cells to investigate the effect of XPO1 on cholangiocarcinoma cell proliferation (Figure 5A). CCK‐8 assays showed that overexpression of XPO1 significantly promoted the proliferation of cholangiocarcinoma cells (Figure 5B,C). In addition, the colony formation assay indicated that XPO1 overexpression significantly increased the number and size of colonies formed by cholangiocarcinoma cells (Figure 5D). In addition, the EdU‐488 DNA synthesis assay showed a significant pro‐proliferative effect of KPT‐330 on RBE and 9810 cells (Figure 5E). These results suggest that XPO1 promotes the proliferation of cholangiocarcinoma cells.

### 3.6 Influence of XPO1 inhibitor KPT‐330 on p53

**FIGURE 6:**
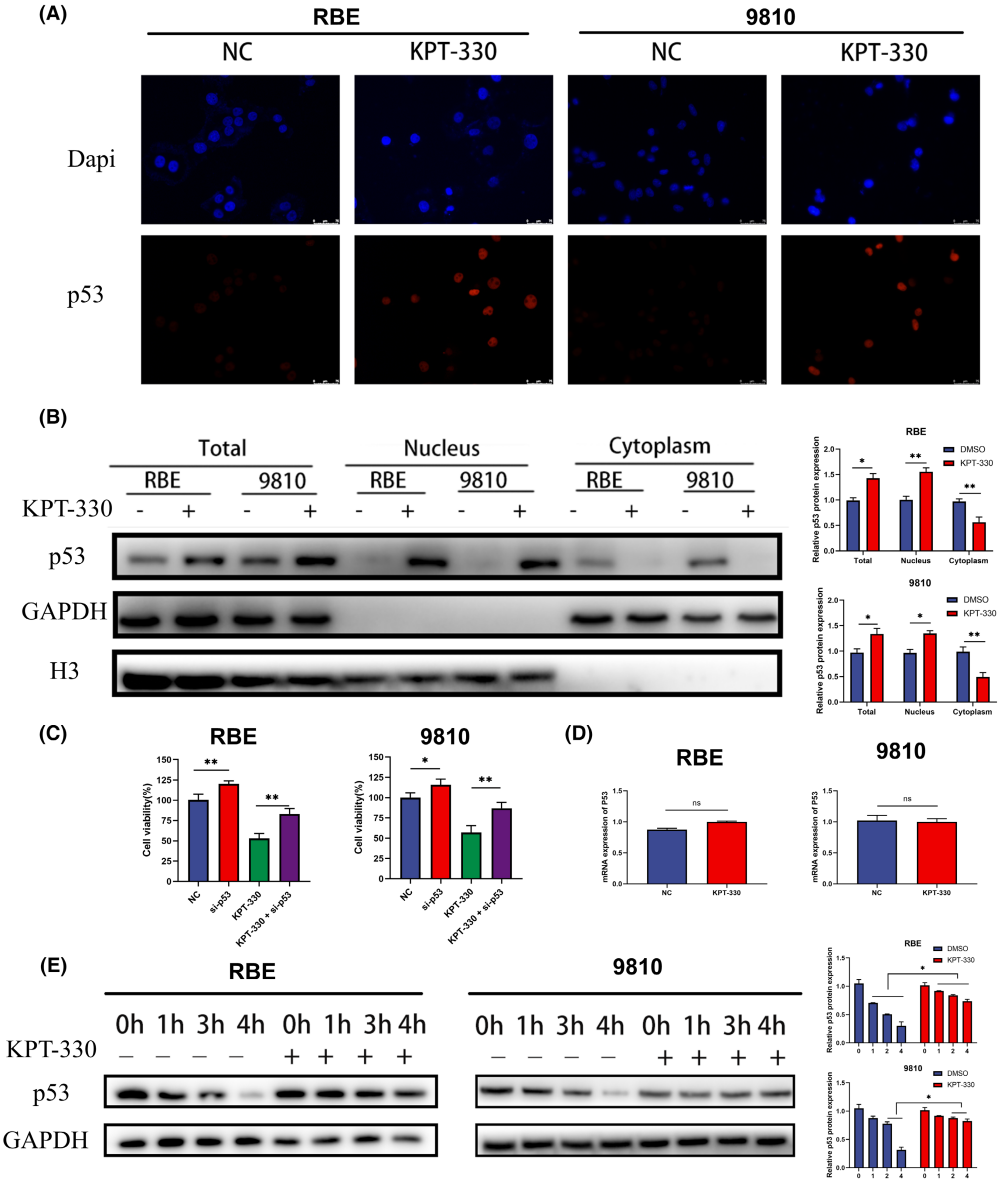
Influence of exportin‐1 (XPO1) inhibitor KPT‐330 on p53. (A) Immunofluorescence assay of p53. (B) Western blot results of the nucleus and cytoplasmic components after KPT‐330 treatment. (C) Cell proliferation of RBE and 9810 under KPT‐330 treatment and p53‐siRNA. (D) qRT‐PCR analysis of p53 mRNA under KPT‐330 treatment. (E) Western blot of the degradation rate of p53 protein after KPT‐330 treatment. Data were shown as mean ± SD. *p < 0.05, **p < 0.01, ***p < 0.001, ****p < 0.0001.

p53 is a carrier protein for XPO1.^12^, ^13^ Therefore, we conducted an immunofluorescence assay to clarify p53 status under KPT‐330 treatment. The results showed that XPO1 inhibition by KPT‐330 triggered the accumulation of p53 inside the nucleus (Figure 6A). Next, we analyzed the changes in the nuclear and cytoplasmic levels of p53 using a western blot, and the results suggested that p53 accumulated in the nucleus after KPT‐330 treatment (Figure 6B). Knockdown of p53 using siRNA rescued the inhibitory effect of KPT‐330 (Figure 6C). This indicates that XPO1 inhibition by KPT‐330 could activate the p53 function to exert anticancer effects. Furthermore, we found that the mRNA expression of p53 did not change significantly under the KPT‐330 treatment (Figure 6D). Next, we blocked the translation of p53 with CHX and detected protein expression of p53 at four time points: 0, 1, 2, and 4 h. Western blot results showed that KPT‐330 significantly reduced the degradation rate of p53 protein in RBE and 9810 cells (Figure 6E). In summary, XPO1 inhibition triggered the accumulation of p53 in the nucleus and improved its stability.

### 3.7 Effect of BIRC6 on p53

**FIGURE 7:**
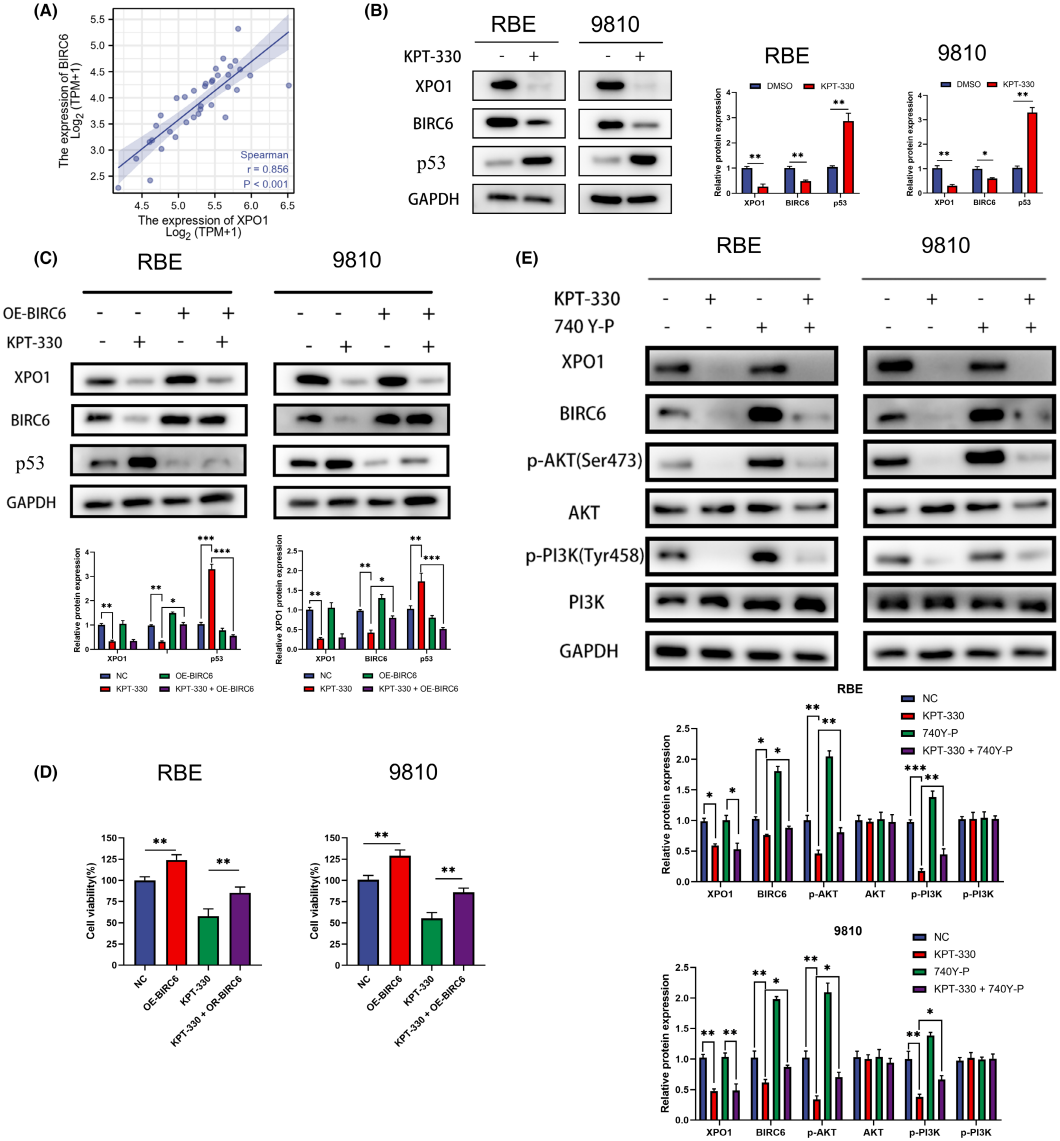
Effect of BIRC6 on p53. (A) Correlation between exportin‐1 (XPO1) and BIRC6 from TCGA. (B) Western blot of p53 and BIRC6 under KPT‐330 treatment. (C) Western blot of p53 and BIRC6 under KPT‐330 treatment and overexpression of BIRC6. (D) Cell proliferation of RBE and 9810 under KPT‐330 treatment and overexpression of BIRC6. (E) Western blot of PI3K/AKT pathway and BIRC6 under KPT‐330 treatment and 740 Y‐P. Data were shown as mean ± SD. *p < 0.05, **p < 0.01, ***p < 0.001, ****p < 0.0001.

Based on previous bioinformatics analysis, we screened the protein BIRC6, which may affect the progression of cholangiocarcinoma cells with XPO1. We first analyzed the correlation between XPO1 and BIRC6. The result suggested that the expression of BIRC6 was closely related to that of XPO1 (Figure 7A). Tang et al. reported that BIRC6 facilitates p53 degradation in hepatocellular carcinoma.^14^ We detected the expression of BIRC6 and p53 after KPT‐330 treatment. Western blot results showed that BIRC6 expression was significantly decreased; however, p53 expression was significantly increased under KPT‐330 treatment (Figure 7B). Next, we overexpressed BIRC6 and assessed its effect on p53 expression. The results indicate that the overexpression of BIRC6 reduced the increase in p53 caused by XPO1 inhibition by KPT‐330 (Figure 7C). Furthermore, we used CCK‐8 to assess the effect of BIRC6 overexpression on the efficacy of KPT‐330. The results in Figure 7D show that overexpression of BIRC6 rescued the inhibitory effect on the proliferation of KPT‐330. XPO1 inhibition has been reported to inhibit the PI3K/AKT pathway, as demonstrated in our study (Figure 7E). Furthermore, we found that the addition of the PI3K agonist 740 Y‐P rescued the reduction in BIRC6 caused by XPO1 inhibition. Overall, XPO1 inhibition reduced the expression of BIRC6 by inhibiting the PI3K/AKT pathway and thus reducing p53 degradation.

## 4 DISCUSSION

XPO1 carries NES‐containing cargo and transports it from the nucleus to the cytoplasm, thus playing an important role in maintaining cell survival.^15^, ^16^ XPO1 dysfunction leads to the mislocalization of cargo proteins, including many proteins associated with tumor progression, such as p53 and p27.^17^ In normal cells, tumor suppressors function in the nucleus, but in tumor cells, XPO1 is overexpressed and transports tumor suppressors out of the nucleus, thereby promoting tumor progression.^18^, ^19^

We found that XPO1 was highly expressed in 21 tumor types, including BLCA, BRCA, CESC, CHOL, COAD, DLBC, ESCA, GBM, HNSC, KICH, KIRP, LAML, LGG, LIHC, LUAD, LUSC, PAAD, READ, STAD, TGCT, and THYM. In terms of prognosis, high XPO1 expression was significantly associated with overall survival and progress‐free interval in LIHC and PAAD. Although similar results were not observed in CHOL, high expression of XPO1 was significantly associated with the “N stage” and “vascular invasion” in CHOL. However, we found that the high expression of XPO1 was significantly associated with overall survival in patients with CHOL in the GEO database. These results suggest that high XPO1 expression is associated with the prognosis of patients with cholangiocarcinoma.

Using the R package clusterProfiler, GO enrichment analysis and KEGG pathway analysis were performed on the 500 genes positively associated with XPO1 expression in CHOL. GO functional enrichment analysis indicated that XPO1 was related to “modified and binding function,” and KEGG analysis revealed that genes involved in the spliceosome, RNA transport, ubiquitin‐mediated proteolysis, and cell cycle terms were significantly enriched. These results are similar to the mRNA‐seq results for neuroblastoma reported by Li‐jia Pan et al.^7^ From the above analysis, we concluded that XPO1 is significantly associated with multiple tumor pathways in CHOL.

Furthermore, we found that inhibition of XPO1 significantly inhibited tumor cell proliferation.^20^, ^21^, ^22^ Leptomycin B, the first specific XPO1 inhibitor, was discovered in the 1990s and has a hyperspecificity for XPO1.^23^ However, its high toxicity limits its clinical application.^24^ With time, several selective inhibitors of nuclear export (SINEs) have been developed. KPT‐330 (Selinexor), a novel oral SINE, has been approved by the United States Food and Drug Administration for the treatment of refractory multiple myeloma and relapsed/refractory diffuse large B‐cell lymphoma. Therefore, we selected KPT‐330 as an inhibitor of XPO1 for the subsequent experiments. The results showed that the XPO1 inhibitor KPT‐330 significantly disrupted CHOL cell proliferation.

p53 acts as a carrier protein for XPO1, and it naturally accumulates in the nucleus upon XPO1 inhibition. We demonstrated that KPT‐330 affects the proliferation of cholangiocarcinoma cells through the nuclear accumulation of p53. Based on the results of bioinformatics analysis, we noted that XPO1 might be associated with protein degradation; therefore, further experiments demonstrated that the XPO1 inhibitor KPT‐330 increased the stability of p53. We unearthed the key gene BIRC6, which can cause the degradation of p53.^14^ BIRC6 carries an N‐terminal single baculovirus inhibition of apoptosis protein repeat (BIR) domain and a C‐terminal ubiquitin‐conjugating (UBC) enzyme domain, while through the UBC domain BIRC6 facilitates proteasomal degradation.^25^ However, the XPO1 inhibitor KPT‐330 reduced the expression of BIRC6, which also explained how XPO1 inhibitor KPT‐330 increased p53 protein stability. The PI3K/AKT pathway plays an important role in the regulation of several cellular processes, including the maintenance of proliferative signaling.^26^ We found that XPO1 inhibitor KPT‐330 inhibited the PI3K/AKT pathway and further inhibited BIRC6 expression.

## 5 CONCLUSION

**FIGURE 8:**
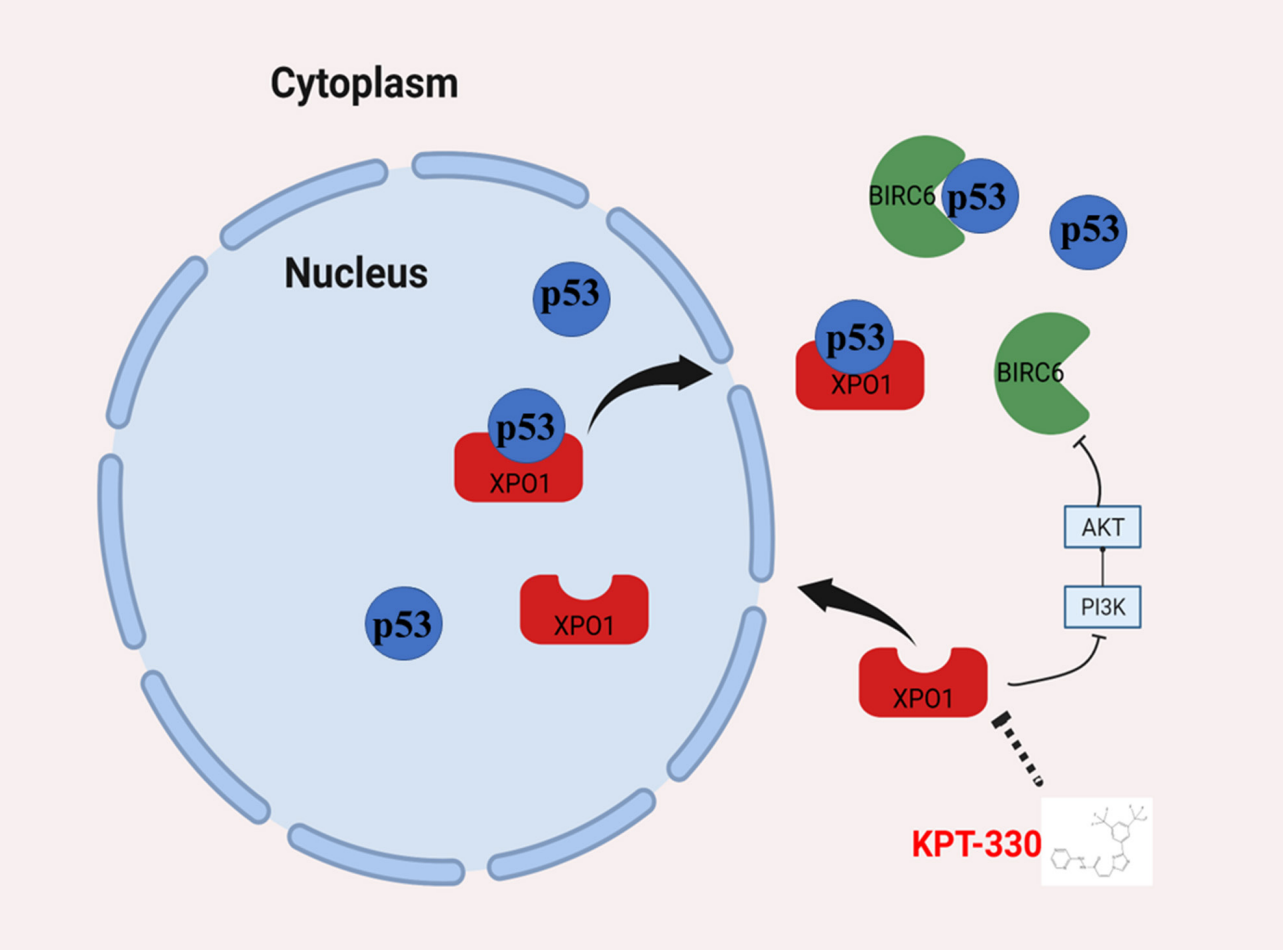
Schematic diagram of effects of XPO1 inhibition by KPT‐330 in CHOL cells. CHOL, cholangiocarcinoma; XPO1, exportin‐1.

In conclusion, we analyzed data from TCGA and found that XPO1 showed high expression across cancers and was associated with poor prognosis in hepatobiliary and pancreatic tumors. In CHOL, we revealed that XPO1 inhibitor KPT‐330 triggered the nuclear accumulation of the p53 protein and significantly disrupted the proliferation of cholangiocarcinoma cells. Mechanistically, XPO1 inhibitor KPT‐330 reduced BIRC6 expression by inhibiting the PI3K/AKT pathway to decrease p53 degradation and improve its stability (Figure 8). Therefore, XPO1 may be a potential therapeutic target, and KPT‐330 may play a therapeutic role in cholangiocarcinoma.

## AUTHOR CONTRIBUTIONS

**Cheng Zhao:** Conceptualization (lead); data curation (lead); formal analysis (lead); investigation (lead); project administration (lead); software (lead); validation (lead); visualization (lead); writing – original draft (lead); writing – review and editing (lead). **Ben Ma:** Conceptualization (equal); data curation (equal); investigation (equal); software (equal); supervision (equal); validation (equal); writing – original draft (supporting). **Ziyi Yang:** Conceptualization (equal); data curation (equal); formal analysis (equal); investigation (equal); methodology (equal); project administration (equal). **Ou Li:** Conceptualization (equal); methodology (equal); resources (equal); software (equal). **Shilei Liu:** Supervision (equal); visualization (equal). **Lijia Pan:** Data curation (equal); formal analysis (equal); funding acquisition (equal). **Wei Gong:** Conceptualization (equal); data curation (equal); formal analysis (equal); funding acquisition (equal); supervision (equal).

## FUNDING INFORMATION

This study was supported by the National Natural Science Foundation of China (nos. 82172628, 81974371, 31701108, 82002503, and 82173081), The Shanghai Sailing Program (nos. 20YF1430200 and 22YF1427300), The Shanghai Anticancer Association Eyas Program (SACA‐CY21B05), and Shanghai scientific and technological innovation plan (22S31903600).

## CONFLICT OF INTEREST

The authors have no conflict of interest to disclose.

## Data Availability

The data sets presented in this study can be found in online repositories. The names of the repository/repositories and accession number(s) can be found in the article.
